# Correlates of viral suppression among sexual minority men and transgender women living with HIV in Mpumalanga, South Africa

**DOI:** 10.1371/journal.pgph.0003271

**Published:** 2024-07-22

**Authors:** Akua O. Gyamerah, Alexander Marr, Kabelo Maleke, Albert E. Manyuchi, Ali Mirzazadeh, Oscar Radebe, Tim Lane, Adrian Puren, Wayne T. Steward, Helen Struthers, Sheri A. Lippman

**Affiliations:** 1 Division of Prevention Science, Department of Medicine, University of California, San Francisco, San Francisco, California, United States of America; 2 Institute for Global Health Sciences, University of California, San Francisco, San Francisco, California, United States of America; 3 Anova Health Institute, Johannesburg, South Africa; 4 National Institute for Communicable Diseases, Johannesburg, South Africa; 5 Division of Virology School of Pathology, University of the Witwatersrand Medical School, Johannesburg, South Africa; 6 Division of Infectious Diseases & HIV Medicine, Department of Medicine, University of Cape Town, Cape Town, South Africa; The Chinese University of Hong Kong, HONG KONG

## Abstract

Sexual minority men (SMM) and transgender women in South Africa engage in HIV care at lower rates than other persons living with HIV and may experience population-specific barriers to HIV treatment and viral suppression (VS). As part of a pilot trial of an SMM-tailored peer navigation (PN) intervention in Ehlanzeni district, South Africa, we assessed factors associated with ART use and VS among SMM at trial enrolment. A total of 103 HIV-positive SMM and transgender women enrolled in the pilot trial. Data on clinical visits and ART adherence were self-reported. VS status was verified through laboratory analysis (<1000 copies/ml). We assessed correlates of VS at baseline using Poisson generalized linear model (GLM) with a log link function, including demographic, psychosocial, clinical, and behavioral indicators. Among participants, 52.4% reported ART use and only 42.2% of all participants had evidence of VS. Of the 49.5% who reported optimal engagement in HIV care (consistent clinic visits with pills never missed for ≥ 4 consecutive days) in the past 3-months, 56.0% were virally suppressed. In multivariable analysis, SMM were significantly more likely to be virally suppressed when they were ≥ 25 years of age (Adjusted prevalence ratio [APR] = 2.0, CI 95%:1.0–3.8); in a relationship but not living with partner, as compared to married, living together, or single (APR = 1.7, CI 95%:1.0–2.7), and optimally engaged in care (APR = 2.1, 95% CI:1.3–3.3). Findings indicate a need for targeted treatment and care support programming, especially for SMM and transgender women who are young and married/living with their partners to improve treatment outcomes among this population.

## Introduction

The proportion of people living with HIV (PLHIV) who have initiated antiretroviral therapy (ART) and have achieved viral load suppression in sub-Saharan Africa remains lower than the 95-95-95 HIV targets despite efforts to increase linkage and retention in ART over the past decade [1]. In South Africa, where good progress has been made on these targets, 90% of PLHIV are aware of their HIV status, 91% of those are on ART, and 94% of PLHIV on ART virally suppressed [2], up from 85%, 71%, and 87% respectively in 2017 [3]. However, for sexual minority men (SMM; i.e., gay, bisexual, queer, men who have sex with men, and other same gender loving men), a population with a 29.7% HIV prevalence—the proportion on treatment is much lower, with 46% on ART and of those 93% achieving viral suppression [4] up from 31.1% and 26.5% respectively in 2018 [5]. For sexually active transgender women, estimates from a surveillance study between 2018 to 2019 indicates a wider disparity, with HIV prevalence ranging from 43.7% to 74.1% and only 24.2% to 54.2% reporting awareness of their HIV-positive status [6]. Among those who know their status, 64.7% to 82.1% are on ART, while 34.4% to 55.0% are virally suppressed [6]. Recent estimates indicate better progress towards 95-95-95 targets—an improvement that is largely due to South Africa’s implementation of the universal “test-and-treat” policy in 2016, which gave access to treatment for all PLHIV, and the introduction of single dose regimens [7, 8]. However, HIV treatment progress for SMM and transgender women in South Africa is suboptimal [4].

Previous research indicates that there are numerous individual, social, structural, and psychosocial barriers that prevent timely and consistent uptake of ART in sub-Saharan Africa and ultimately, viral suppression among PLHIV. These factors include clinic accessibility, HIV stigma, health systems barriers, and fear of ART side effects [9]. Younger age, substance abuse, and lack of social support have been reported as barriers to engagement in care and viral suppression for PLHIV [10–13]. For SMM, factors such as active participation in a gay community, treatment accessibility, and self-efficacy improve ART uptake and retention in care [14, 15], while lack of SMM-friendly clinics, substance abuse, and lack of psychosocial support are barriers to ART adherence [16].

A 2017 study reporting viral suppression data among SMM and transgender individuals in Johannesburg, South Africa found that among those living with HIV (n = 118), 46.9% were virally suppressed and older age and buying sex from men in the past year were associated with viral suppression [17]. However, overall, few estimates of HIV viral suppression among SMM and transgender women exist in Southern Africa, especially in rural areas. Moreover, few SMM and transgender cohorts exist to understand factors contributing to poor engagement and viral suppression to inform responses for a population facing high intersectional stigma.

To address this research gap and the disparity in HIV treatment outcomes among SMM and transgender women in the region, evidence-based interventions are needed that address the specific care needs of this population. Peer navigation (PN) is one such intervention that has been found to improve linkage and retention in care among SMM of color in the U.S. [18] and in PLHIV attending clinics in the North West Province of South Africa [19, 20]. We, therefore, developed the Bontsanga Bokuphila (Peers for Life) study, a pilot trial of an SMM-tailored PN intervention to improve HIV care engagement for HIV-positive sexual and gender minorities in rural South Africa (https://clinicaltrials.gov/study/NCT03483857). This paper characterizes the cohort’s (N = 103) ART uptake, engagement in HIV care, and viral suppression status at baseline in order to assess factors influencing treatment successes in this key population prior to the trial.

## Materials and methods

### Study design and setting

The present study reports baseline cross-sectional data from a peer navigation randomized control trial. The baseline survey was conducted between 16 September and 31 October 2017 in the Ehlanzeni District of Mpumalanga, South Africa, a largely rural region where HIV is a leading cause of death with adult prevalence at 11.7% [21]. Ehlanzeni is home to a significant and diverse SMM community. In a previous study among SMM in the district, our team documented HIV prevalence at 13.7%, with only 11.3% linked to care and 9.6% initiated on ART [22].

The study office was located in Mbombela, Ehlanzeni district. The recruitment area included all Ehlanzeni sub-districts where study the partner, Anova Health Institute, had established Health4Men—a comprehensive HIV intervention program implemented in partnership with the Provincial Department of Health. To ensure access to a non-stigmatizing clinical environment for participants, a Regional Leadership Site (RLS) and SMM-friendly clinical sites were established to serve as referral centers for HIV care.

### Recruitment procedures

The parent study enrolled a total of 103 participants, who were recruited from two sources: newly diagnosed HIV-positive SMM at Ehlanzeni district clinics and from two previous NIH-supported studies for which participants had consented to be contacted for future research. These two studies were the Mpumalanga Men’s Study (R01AI089292), which diagnosed 240 HIV-positive SMM in Ehlanzeni [22, 23], and a partnering HIV self-testing study (R21MH103038), which identified 18 SMM [24]. Potential participants from these studies were screened by phone to assess interest and eligibility. For the clinic-based recruitment, we worked with 5 SMM-friendly partnering clinics and the RLS to recruit newly diagnosed SMM or SMM who presented for confirmatory testing/CD4 blood draw. HIV test counselors at the partnering clinics asked SMM testing positive for HIV or returning for HIV staging if they would be interested in participating in the research study. When a participant was interested, the test counselor recorded their preferred method of contact and let the potential participant know that study staff would be contacting them for eligibility screening. We did not enroll SMM who were already established in care at the partnering clinics as they were unlikely to require navigation services. All eligible participants provided written informed consent in the language of their choice–Siswati, English, or IsiZulu–prior to study participation. Of the 103 participants, 62 were recruited from previous study participants and 41 from partnering clinics.

Study eligibility criteria were: assigned male at birth; identify as gay/bisexual, a transgender woman, or a man who has sex with men (MSM); self-reported sex with a man within six months prior to recruitment; HIV-positive verified by test; 18 years or older, and resident of Ehlanzeni. The term “men who have sex with men” was used as a behavioral term for recruitment, rather than as an identity category. In this study, we use the term “sexual minority men” to encompass gay, bisexual, queer, MSM, and other same gender loving men. While the study focused on SMM, about 10.7% (n = 11) of participants identified as transgender women. Treatment status was not an eligibility criterion.

### Data collection

Behavioral data were obtained through a survey administered in English, SiSwati, or IsiZulu at baseline. Questions included socioeconomic characteristics, sexual/gender identity, psychosocial wellbeing, sexual history/activities, ART initiation/adherence, and HIV care history. Computer assisted, interviewer-administered interviews using QDS (Nova Research, Bethesda, MD) were conducted by trained bilingual personnel in a private room at the study site. After completing the baseline questionnaire, participants were randomized to receive either standard-of-care referral to SMM-competent HIV care or referral plus peer navigation treatment support. Participants were reimbursed 150 South African Rand ($10.50) per assessment visit.

Laboratory procedures: Consenting participants were tested by a trained nurse; Dried Blood Spot (DBS) samples were sent to the National Institute of Communicable Diseases (NICD) for laboratory testing. The NICD confirmed the HIV-positive status of samples using a 3^rd^ -generation HIV ELISA (Genscreen^™^ HIV1/2 version 2, Bio-Rad, Marnes-la-Coquette, France). Reactive ELISA results were confirmed using a 4^th^-Generation ELISA (Vironostika^™^, HIV Ag/Ab AssayI, bioMérieux, Marcy-I’Etoile, France). Viral load testing was conducted using the Abbott m2000 HIV Real-Time System (Abbott Molecular Inc., Des Plaines, Illinois, USA).

### Key measures

Viral suppression (VS)—the main outcome of interest—was assessed at enrolment and defined as undetectable viral load or under 1000 copies/ml. Demographic indicators included gender identity/expression [25], sexual orientation, race and ethnicity, age, education, employment, living situation, marital status, and monthly income. Behavioral indicators included alcohol use, utilizing the AUDIT-C, with a score of ≥ 4 interpreted as a sign of alcohol misuse [26].

We measured anticipated HIV stigma using an adapted 9-item validated scale [27, 28] inquiring whether participants anticipated negative consequences in response to disclosing positive status (e.g, “You would lose your job/ livelihood”; Cronbach alpha = 0.86). Responses were dichotomized into any and no anticipated stigma. We measured enacted stigma using an adapted 11-item validated scale to assess participants’ experiences of stigma events due to their HIV-status (e.g., “People treated me with less respect because I am HIV-positive”; Cronbach alpha = 0.84). We dichotomized responses into any and no enacted stigma. Barriers to accessing healthcare were assessed using a 16-item scale (e.g., “You have problems remembering appointments” and “You feel too depressed to go to the clinic”) modified from the Barriers and Facilitators scale [29] and coded as any barrier or no barriers (Cronbach alpha = 0.72). Finally, mental health was assessed using a 10-point scale (e.g., “you felt lonely” and “your sleep was restless”; Cronbach alpha = 0.86) adapted from the Centers for Epidemiological Survey Depression Scale [30]. Responses were dichotomized into any indicator of depression or not experiencing depression symptoms.

Clinical indicators: Participants were asked about date of HIV diagnosis and ART and CD4/viral load testing history. They also reported their ART uptake, treatment history, and engagement in HIV care ≤ 3 month to assess HIV treatment adherence and care engagement. All measures of ART adherence and HIV care engagement were self-reported. Optimal engagement in HIV care was defined as self-reported adherence to clinical visits with self-reported adherence to ART including never missing ART for four days or more in a row over the past three months [31].

### Analysis

Analyses were conducted using STATA version 15.1 (College Station, TX). Participant characteristics at baseline were summarized using means, medians and interquartile ranges for continuous variables and proportions and frequencies for categorical variables. Bivariate analyses were conducted to examine correlates of viral suppression using chi-square tests for categorical variables and Wilcoxon Rank Sum tests for continuous variables, with statistical significance set at *P*<0.05.

To isolate the predictors of viral suppression, we used multivariable regression using a generalized linear model (GLM) with a Poisson distribution and log link function. This provides adjusted prevalence ratios (PR) as a measure of association along with 95% confidence intervals (CI). The regression models were built using significant variables from bivariate analyses and theoretically relevant variables based on extant literature to examine independent predictors of viral suppression. Multivariate models were adjusted for age. Variables explored included: age, income, marital status, HIV diagnosis year, engagement in care, ART use, optimal engagement, barriers to care, anticipated HIV stigma, ART counselling at last clinic visit, alcohol use, and HIV disclosure status. The following variables were not included in the model due to excessive missing values (> 12% of observations): mental health, ART counselling at last clinic visit, engagement in HIV care ≤ 3 months, number of clinic visits in past year, and years since ART initiation.

### Ethical approval and consent to participate

The study was approved by the Institutional Review Board of the University of California, San Francisco (IRB # 17–22752) and the Human Research Ethics Committee at the University of the Witwatersrand in South Africa (Certificate # M170533). All participants provided written informed consent before study participation.

## Results

Out of the 120 people assessed for eligibility, 103 eligible participants enrolled in the study. Table 1 presents demographic, behavioral, psychosocial, and clinical characteristics of the sample. The mean age was 30.9 years, 41.8% of participants identified as gay and 10.7% identified as trans women. Heavy alcohol use (79.6%) was common. Notably, a number of respondents did not recall their clinical history; between 14.0%-26.0% have missing values for HIV diagnosis date, number of clinical visits in past year, HIV care visit within the past 3 months, and whether they received ART counselling at their last visit.

**Table 1:** Demographic, behavioral, psychosocial, and clinical characteristics of SMM cohort at baseline, N = 103.

Variable	N (%)
**Demographics**	
**Age** (Mean = 30.9, SD = 9.4)	
18–24	27 (26.2)
25–34	48 (46.6)
35+	28 (27.2)
**Gender**	
Male	92 (89.3)
Trans woman	11 (10.7)
**Gender expression**	
Feminine	31 (30.1)
Masculine	72 (69.9)
**Sexual identity**	
Gay/homosexual	43 (41.8)
Bisexual	42 (40.8)
Straight/heterosexual	9 (8.7)
Transgender/woman	9 (8.7)
**Marital Status**	
Single	44 (42.7)
In relationship but not living with partner	38 (36.9)
Married/living with partner	21 (20.4)
**Educational attainment**	
Primary or less	19 (18.5)
Some or completed secondary	68 (66.0)
College, university, technical	16 (15.5)
**Income per month**	
Low income (R0-R1199)	64 (62.8)
Middle income (R1200-R2499)	15 (14.7)
High income (R2500-R9999)	23 (22.6)
**Behavioral & Psychosocial**	
**Heavy alcohol use in past 6 months**	82 (79.6)
**Drug use in past 6 months**	18 (17.5)
**Any anticipated HIV stigma**	55 (53.4)
**Any enacted HIV stigma** (n = 102)	62 (60.8)
**Disclosed HIV status to anyone outside of clinic**	65 (65.0)
**Disclosed HIV status to partner/spouse**	47 (45.6)
**Number of mental health issues reported,** Median (IQR) (n = 65)	2 (2, 3)
**Number of barriers to healthcare services,** Median (IQR) (n = 99)	0 (0,2)
**Clinical**	
**Number of clinic visits in past 12 months.,** Median (IQR) (n = 89)	4 (2, 12)
**Years since HIV diagnosis,** Mean (SD) (n = 78)	2.0 (3.0)
**Visited clinic for HIV care in last 3 mos.** (n = 77)	73 (94.8)
**Ever received ART**	56 (54.4)
**Currently using ART**	54 (52.4)
**Years since ART started, Mean (SD) (n = 51)**	1.9 (2.3)
**Not missed medication in last month (n = 54)**	41 (75.9)
**Not missed ART 4 days in a row in last 3 mos. (n = 54)**	46 (85.2)
**Optimally engaged in HIV care** **	51 (49.5)
**Received ART counseling at last HIV clinic visit** (n = 76)	61(80.3)
**Disclosed as SMM at last clinic visit** (n = 86)	32 (37.2)
**Viral suppression** (<1000 copies/ml) (n = 102)	43 (42.2)

*Percentages expressed of those with non-missing responses.

**Adhered to ART and engaged in care in last 3 mos

About half of the participants with clinical history responses were on ART; 49.5% were identified as optimally engaged in HIV care (Table 1). About 65.0% of participants reported disclosing their HIV status to someone outside of a clinical setting and 60.8% reported experiencing stigma following disclosure.

### Correlates of viral suppression

Viral load testing indicated that 42.2% (95% CI: 32.4% -52.3%) of participants were virally suppressed (<1000 copies/ml) at baseline. Table 2 presents data on the bivariate associations of viral suppression and demographic, behavioral, psychosocial, and clinical indicators. Those who were age 25 years and older compared to younger participants (48.0% v. 25.9%; χ^2^ = 4.0, p = 0.05) and those optimally engaged in care compared to those not (56.0% vs. 28.8%; χ^2^ = 7.7, p = 0.01) were more likely to be virally suppressed. Viral suppression was also less likely among participants who visited the clinic less frequently (z = -3.008; p = 0.003); in other words, the median clinic visits among those who were not virally suppressed was significantly lower than the median clinic visits of those virally suppressed. Participants who initiated ART earlier (Mean = 2.7 vs. 1.0; p = 0.01) and who received counselling on taking/adhering to ART (52.5% vs. 21.4%; χ^2^ = 4.4, p = 0.04) were also more likely to be virally suppressed. Participants who had disclosed their HIV status to their partner/spouse versus not (52.2% vs. 33.9%, *χ*^2^ = 3.4; p = 0.06) were more likely to be virally suppressed though it was not significant.

**Table 2:** Bivariate associations of viral suppression and demographic, behavioral, psychosocial, and clinical indicators, N = 102.

Variable	Viral Suppression, n (%)			*P-value*
	No (n = 59)	Yes (n = 43)	*χ* ^2^	
**Age**				
18–24	20 (74.1)	7 (25.9)	4.0	0.05
25 and older	39 (52.0)	36 (48.0)		
**Gender**				
Male	53 (58.2)	38 (41.8)	0.1	1.0
Transwoman	6 (54.6)	5 (45.4)		
**Sexual identity**				
Gay/homosexual	25(58.1)	18 (41.9)	2.5	0.53
Bisexual	21 (51.2)	20 (48.8)		
Straight/heterosexual	7 (77.8)	2 (22.2)		
Transgender/woman	6 (66.7)	3 (33.3)		
**Marital Status**				
Single	29 (65.9)	15 (34.1)	2.2	0.33
In relationship but not living with partner	19 (50.0)	19 (50.0)		
Married/living with partner	11 (55.0)	9 (45.0)		
**Educational Attainment**				
Primary or less	13 (72.2)	5 (27.8)	2.3	0.31
Some or completed secondary	36 (52.9)	32 (47.1)		
College, university, technical	10 (62.5)	6 (37.5)		
**Income per month**				
Low income (R0-R1199)	38 (59.4)	26 (40.6)	0.4	0.88
Middle income (R1200-R2499)	8 (57.1)	6 (42.9)		
High income (R2500-R9999)	12 (52.2)	11 (47.8)		
**Heavy alcohol use in past 6 months**				
Yes	48 (58.5)	34 (41.5)	0.1	0.77
No	11 (55.0)	9 (45.0)		
**Drug use in past 6 months**				
Yes	11 (61.1)	7 (38.9)	0.1	0.80
No	48 (57.1)	36 (42.9)		
**Any anticipated HIV stigma**				
Yes	31 (56.4)	24 (43.6)	0.1	0.74
No	28 (59.6)	19 (40.4)		
**Any enacted HIV stigma**				
Yes	24 (60.0)	16 (40.0)	0.1	0.79
No	35 (57.4)	26 (42.6)		
**Disclosed HIV status to anyone outside of clinic**				
Yes	34 (53.1)	30 (46.9)	1.5	0.23
No	23 (65.7)	12 (34.3)		
**Disclosed HIV status to partner/spouse**				
Yes	22 (47.8)	24 (52.2)	3.4	0.06
No	37 (66.1)	19 (33.9)		
**Number of mental health issues reported,** Median (IQR)^+^	2 (2, 5)	2 (2, 2)	z = 1.930	0.05
**Experienced any barriers to health services**				
Yes	30 (63.8)	17 (36.2)	1.2	0.28
No	27 (52.9)	24 (47.1)		
**Number of clinic visits in past 12 months,** Median (IQR)^+^	3 (2, 9)	6 (3, 12)	z = -3.008	0.003
**Years since HIV diagnosis,** Mean (SD)^+^	1.4 (2.6)	2.6 (3.3)	z = -1.665	0.1
**Visited clinic for HIV care in last 3 mos.**				
Yes	37 (51.4)	35 (48.6)	0.8	0.62
No	3 (75.0)	1 (25.0)		
**Ever received ART**				
Yes	24 (43.6)	31 (56.4)	10.3	0.001
No	32 (76.2)	10 (23.8)		
**Currently using ART**				
Yes	24 (45.3)	29 (54.7)	7.0	0.01
No	31 (72.1)	12 (27.9)		
**Years since ART started, Mean (SD)** ^+^	1.0 (1.0)	2.7 (2.8)	_	0.01
**Not missed ART 4 days in a row in last 3 mos.**				
Yes	21 (46.7)	24 (53.3)	0.2	0.7
No	3 (37.5)	5 (62.5)		
**Optimally engaged in HIV care** **				
Yes	22 (44.0)	28 (56.0)	7.7	0.01
No	37 (71.2)	15 (28.8)		
**Received ART counseling at last HIV clinic visit**				
Yes	29 (47.5)	32 (52.5)	4.4	0.04
No	11 (78.6)	3 (21.4)		
**Disclosed as SMM at last clinic visit**				
Yes	20 (62.5)	12 (37.5)	1.1	0.30
No	27 (50.9)	26 (49.1)		

* Percentages expressed of those with non-missing responses

^+^Indicates Wilcoxon Rank Sum test used

**Adhered to ART and engaged in care in last 3 mos.

### Multivariate associations

In multivariate analysis, independent significant correlates of viral suppression were age 25 years and older (APR = 2.0, 95% CI: 1.0–3.8), in a relationship but not living with partner (APR = 1.7, 95% CI: 1.0–2.7), and optimal engagement in HIV care (APR = 2.1, 95% CI: 1.3–3.3) (Table 3).

**Table 3:** Correlates of viral suppression, N = 102.

Variable	Crude PR	Adjusted PR (95% CI)	*P-value*
Model			
**Age**			
18–24 years	1.0 [Ref]	1.0 [Ref]	1.0 [Ref]
25 years and older	1.9 (0.9–3.7)	2.0 (1.0–3.8)	0.04
**Marital status**			
Single or widowed	1.0 [Ref]	1.0 [Ref]	1.0 [Ref]
Married or living with partner	1.3 (0.7–2.5)	1.0 (0.5–1.9)	1.0
In relationship but not living with partner	1.5 (0.9–2.5)	1.7 (1.0–2.7)	0.04
**Optimally engaged in HIV care** *	1.9 (1.2–3.2)	2.1 (1.3–3.3)	0.002

* Adhered to ART and engaged in care in last 3 mos

## Discussion

We found that a low proportion of South African sexual minority men and transgender women participating in the Bontsanga Bokuphila study were optimally engaged in HIV care and virally suppressed, adding to the limited data currently available to track UNAIDS’ 95-95-95 treatment targets among SMM and transgender women in the region. Our results contribute to the growing understanding of factors shaping HIV care, treatment adherence, and viral suppression among African SMM and transgender women. Our findings can inform targeted programs to address barriers and challenges in linkage, engagement, and retention in treatment among this population that bears a disproportionate burden but remains underserved in HIV care programming.

Over half of the participants had initiated ART, but less than half were optimally engaged in care in the preceding three months. Viral suppression was low among our participants, with only half of those optimally engaged in care and 40% of all participants virally suppressed. While low, the overall prevalence of viral suppression among our sample (40%) is higher than previous reports around the time of our study among SMM (26.5%) in South Africa [5] or Kenya (30.6%) [15], but lower than HIV-positive South African men in the general population (50.8%) [3] and in a study of HIV-positive SMM and transgender people in Johannesburg, South Africa (46.9%) [17]. Our prevalence of 45.4% viral suppression among transgender women also falls within the range reported in a 2018 to 2019 biobehavioral study among transgender women in Johannesburg (34.4%), Cape Town (41.2%), and Buffalo City (55.0%), South Africa [6].

We found that SMM and transgender women who are 25 years and older, optimally engaged, and in a relationship but *not living* with their partner were significantly more likely to be virally suppressed compared to those who were younger, single or married/living with their partner, and not optimally engaged in care. Studies in Africa and other contexts have found optimal engagement to be correlated with viral suppression among PLHIV generally and HIV-positive SMM specifically [14, 32, 33]. Our findings are similar to a study in Kisumu, Kenya in which SMM *not living* with a male partner had higher odds of viral suppression, while SMM younger than 25 years had decreased odds of viral suppression [15]. Similarly, Fearon and colleagues [17] found that older age was associated with viral suppression among a cohort of SMM and transgender people in South Africa. Young SMM and transgender women could be more recently diagnosed, with less time to engage in treatment, and could experience more socioeconomic barriers to care, slowing progress towards viral suppression.

Several South African studies found that unmarried, single, and widowed PLHIV were more likely to take or adhere to ART [34, 35] and a Zambian study noted that married PLHIV were less likely to adhere to ART than divorced/widowed PLHIV [36]. Although counterintuitive, these findings suggest that in low-resource settings being married/living with a partner may create stressors that negatively affects treatment adherence, especially if they are in a heterosexual partnership or face stigma for living in a same-sex marriage. While our study did not distinguish between same- or opposite-sex relationships, future studies should examine whether relationship type and partner gender affect SMM and transgender women’s HIV outcomes.

Consistent with other studies, SMM and transgender women who anticipated HIV stigma were less likely to disclose their status to anyone outside of a clinic [37]. Because anticipated HIV stigma prevents key populations like SMM and transgender women from accessing healthcare services [14, 38, 39], addressing stigma, whether internalized or on the community or structural level, is essential to improving HIV care cascade outcomes. We also found that a majority of participants reported heavy alcohol use and many reported symptoms of depression—factors that have been found to have a negative effect on treatment cascade outcomes [9, 12]. Interventions to address stigma, alcohol use, and emotional well-being among HIV-positive Black South African SMM and transgender women are critically needed.

### Limitations

This study had limited sample size and is not a representative sample of SMM and transgender women living with HIV in the study area; it was designed and powered to assess acceptability and feasibility of a novel peer navigation intervention. With the exception of viral suppression, clinical outcomes including ART adherence and care engagement were self-reported and are thus prone to recall and social-desirability bias, or may just be misreported or underreported due to being poorly understood [40]. In our study, we found that numerous participants did not know or remember aspects of their clinical history. Moreover, substance use have been linked to lower self-reported adherence to treatment and care [41]. Given the high rate of alcohol use and low proportion of optimal engagement and viral load suppression in our sample, it is possible that participants overreported their ART adherence. Finally, Black South African SMM and transgender women who are young, single, or married/living with their partners may face particular barriers to accessing HIV care and achieving viral suppression that were not captured in our study. Future studies should qualitatively examine barriers facing rural Black South African SMM and transgender women living with HIV.

## Conclusions

Our study contributes to the growing literature on factors that shape viral suppression among SMM and transgender women in sub-Saharan Africa. Only one-half of HIV-positive SMM in this study were attending care and virally suppressed—rates far from the 95-95-95 targets and well below the general population. Given the high rate of HIV, low engagement in care, and low attainment of viral suppression among this population, our findings highlight the need to target programs specifically to HIV-positive SMM and transgender women, with particular focus on those who are young, single, and married or living with their partners. Interventions aimed at strengthening treatment and care support by romantic partners, peers, and service providers may improve care engagement and viral suppression. Finally, research is needed to understand why viral suppression remains low, even among those who report ART adherence. These recommended areas of research and intervention can contribute to South Africa’s goal of reducing morbidity and mortality by providing HIV care and adherence support for PLHIV [42].

## Supporting information

S1 Checklist Inclusivity in global research. (DOCX)
